# Free-Breathing and Single-Breath Hold Compressed Sensing Real-Time MRI of Right Ventricular Function in Children with Congenital Heart Disease

**DOI:** 10.3390/diagnostics13142403

**Published:** 2023-07-18

**Authors:** Christoph Treutlein, Martin Georg Zeilinger, Sven Dittrich, Jan-Peter Roth, Matthias Wetzl, Rafael Heiss, Wolfgang Wuest, Matthias Stefan May, Michael Uder, Oliver Rompel

**Affiliations:** 1 Institute of Radiology, University Hospital of Erlangen, Friedrich Alexander University Erlangen-Nürnberg, 91054 Erlangen, Germany; 2 Department of Pediatric Cardiology, University Hospital of Erlangen, Friedrich Alexander University Erlangen-Nürnberg, 91054 Erlangen, Germany; 3 Martha-Maria Hospital, 90491 Nuremberg, Germany

**Keywords:** cardiac magnetic resonance imaging, children, compressed sensing

## Abstract

(1) Purpose: to compare right ventricular (RV) functional parameters in children with surgically repaired congenital heart disease (CHD) using single/double breath hold (BH) and free-breathing (FB) real-time compressed sensing (CS) cine cardiac magnetic resonance (cMRI) with standard retrospective segmented multi breath hold (RMB) cine cMRI. (2) Methods: Twenty patients with CHD underwent BH and FB, as well as RMB cine cMRI, at 3T to obtain a stack of continuous axial images of the RV. Two radiologists independently performed qualitative analysis of the image quality (rated on a 5-point scale; 1 = non-diagnostic to 5 = excellent) and quantitative analysis of the RV volume measurements. (3) Results: The best image quality was provided by RMB (4.5; range 2–5) compared to BH (3.9; range 3–5; *p* = 0.04) and FB (3.6; range 3–5; *p* < 0.01). The RV functional parameters were comparable among BH, FB, and RMB with a difference of less than 5%. The scan times for BH (44 ± 38 s, *p* < 0.01) and FB (24 ± 7 s, *p* < 0.01) were significantly reduced compared to for RMB (261 ± 68 s). (4) Conclusions: CS-FB and CS-BH real-time cine cMRI in children with CHD provides diagnostic image quality with excellent accuracy for measuring RV function with a significantly reduced scan time compared to RMB.

## 1. Introduction

Accurate and reproducible volumetric and functional assessment of the right ventricle (RV) plays an essential role in the management of children with surgically repaired congenital heart disease (CHD) [1,2]. Decisions and strategies in follow-up support are largely based on cardiac magnetic resonance imaging (cMRI) measurements and on changes in RV function captured with repeated cMRI [3]. A number of congenital cardiac conditions predominantly impact the right cardiac chamber, encompassing tetralogy of Fallot, transposition of the great arteries, Ebstein anomaly, and pulmonary atresia. After operative correction, residual anatomic and hemodynamic abnormalities are almost universal. Within these individuals, the long-term risk of impaired functionality of the right ventricle manifests as substantial morbidity and mortality [4]. However, despite increased utilization, cMRI is a physically demanding examination requiring multiple scans and breath hold (BH) commands, resulting in long examination times. It is often limited in children with CHD presenting with dyspnea, difficulty in tolerating BH, or the inability to fully cooperate. Consequently, acquiring diagnostic images frequently requires the utilization of sedation or general anesthesia, posing implications for the safety aspects of MRI [5]. In recent years, there has been growing concern regarding the potential long-term effects of exposure to anesthesia on neurodevelopmental outcomes. This concern is based on preclinical models, as well as retrospective clinical data [6,7,8,9]. Additional mechanical ventilation is necessary for image acquisition using breath hold techniques [10]. This entails potential complications during the intubation process itself, such as tooth or airway injury or aspiration [11]. Furthermore, there are financial considerations to take into account regarding the examination, as well as the impact on the clinical workflow of the radiology department [5]. These factors can add complexity to the overall process and require careful management and resource allocation.

Moreover, the image quality in cMRI greatly depends on heart rate and heart rate variability, thus limiting cMRI, especially in patients with arrhythmias [12]. Therefore, reducing the scan time and the application of real-time sequences is desirable to achieve diagnostic image quality [5]. 

Retrospectively electrocardiogram (ECG)-gated BH cine cMRI is well established and generally considered the reference standard for assessing the RV volume and function with high reproducibility [13,14]. 

At the expense of lower image quality, real-time sequences in the past provided higher temporal resolution compared to that of standard sequences. With recent technical advances and the introduction of techniques such as compressed sensing (CS) reconstruction, it is now possible to acquire the entire heart in a single-shot manner within a single breath hold (SB) or—depending on the number of slices and the patient’s constitution—a double BH command. It is even possible with a free-breathing (FB) technique, with increased spatial resolution and acceptable reconstruction times [15]. 

The left ventricle is routinely assessed using a short-axis orientation. The best orientation for functional assessment of the RV is still debated, but volumes of the RV in a dedicated axial orientation seem to agree more closely with the flow measured in the pulmonary trunk than do volumes acquired with the short-axis orientation [3,16,17].

The aim of this study was to compare standard retrospective segmented multi-breath hold (RMB) cine cMRI of the RV in the dedicated axial orientation with BH and FB compressed sensing real-time cine cMRI regarding image quality and RV function in children with surgically repaired CHD. 

## 2. Materials and Methods

Twenty patients were prospectively included in this intraindividual, comparative study. The patients presented with the following surgically repaired cardiac disorders: tetralogy of Fallot (*n* = 5), coarctatio aortae (*n* = 2), atrioventricular septal defects (*n* = 2), pulmonary artery ectasia or stenosis (*n* = 3), Ebstein’s anomaly (*n* = 3), (multivalvular) vitia (*n* = 2), transposition of the great arteries (*n* = 1), partial anomalous pulmonary venous return (*n* = 1), and Bland–White–Garland syndrome (*n* = 1). There were no specific study inclusion or exclusion criteria in addition to the usual contraindications for cMRI, such as unsafe implants.

The study protocol was approved by the local institutional review board (94_18 B), and the HIPAA (Health Insurance Portability and Accountability Act) criteria were applied. Patients and/or parents were required to give their informed consent for the routine cMRI, as well as for the additional CS sequences obtained. 

### 2.1. CMRI Protocol

CMRI imaging was performed using a 3T MRI system (MAGNETOM Vida, Siemens Healthineers AG, Erlangen, Germany) with dedicated phased-array cardiac receiver coils (18-channel body coil, 72-channel spine coil). The patients were placed in a supine position. Stacks of axial slices covering the entire RV were acquired. A retrospective ECG-gated segmented k-space balanced steady-state free precession pulse sequence (bSSFP, Siemens Medical Solutions) was used as the reference sequence. The aforementioned RMB sequence was performed using the multi-BH technique with one BH per slice at the end of expiration. 

In all patients, prospective, adaptive triggering to the ECG was used for the acquisition of equally planned CS real-time cine cMRI scans using the BH and FB techniques. Detailed imaging parameters are reported in Table 1.

Additionally, T2w half-Fourier acquisition single-shot turbo spin echo imaging (HASTE) and standard retrospective ECG-gated bSSFP sequences were performed on the right and left ventricles, including 4-chamber, 3-chamber, and 2-chamber views; sections across the outflow tracts of the right and left ventricles; and short-axis stack images of the left ventricle. To determine the reference positions for flow measurements, contrast-enhanced MR angiography sequences of the pulmonary arteries and aorta were acquired. 

In all study examinations, phase-contrast flow measurement was performed in the pulmonary artery, positioned midway between the level of the pulmonary valve and the bifurcation of the branch pulmonary arteries, to compare it with the RV stroke volumes (RVSVs), calculated from the standard (RVSVRMB) and CS cine data (RVSVBH and RVSVFB). The presence of larger shunts or valve insufficiencies was excluded by comparing them with phase-contrast flow measurements in the ascending aorta and with the left ventricular stroke volume.

### 2.2. Image Analysis

Analysis was performed by two readers with 18 and 7 years of experience in cardiovascular imaging. Each observer scored and processed all 20 cases in random order while blinded to the diagnoses, patients, sequences, and other imaging data. 

The end systolic and end diastolic phases were detected automatically based on the smallest and largest RV volumes over the entire cardiac cycle [18], and they were verified through visual inspection. Manual segmentation of the endocardial borders of the RV wall on axially oriented RMB, BH, and FB images was performed in all acquired phases of the cardiac cycle using dedicated software (Syngo.via VB30; Siemens Healthineers, Erlangen, Germany), and functional parameters were analyzed. Trabeculation in the myocardial mass and papillary muscles was included in the RV cavity. In the basal slice of the axial data sets—if the pulmonary valve was visible—only the portion of the RV outflow tract below the level of the pulmonary valve was included. For the inflow part of the RV, the blood volume was excluded from the RV volume if the surrounding wall was thin and not trabeculated because it was considered to be in the right atrium [19]. The following parameters were evaluated: right ventricular ejection fraction (RVEF), right ventricular end diastolic volume (RVEDV), right ventricular end systolic volume (RVESV), and right ventricular stroke volume (RVSV). 

The values obtained were indexed for body surface area, calculated according to the Mosteller formula [20]. The acquisition times for the RMB, BH, and FB stacks were obtained from the DICOM time stamps on the first and last images of the stack. 

The subjective image quality was evaluated on a 5-point Likert scale (1 = insufficient, extensive artifact affecting volumetric analysis; 2 = poor, moderate artifacts affecting volumetric analysis; 3 = acceptable, mild artifacts; 4 = good, minimal artifacts not affecting volumetric analysis; 5 = very good, no artifacts) (7). The diagnostic confidence was rated on a 3-point scale (1 = low, 2 = intermediate, 3 = high). 

### 2.3. Statistics

Descriptive statistical data analysis provided the mean values, range, and standard deviation.

Assumptions of normality were checked by the D’Agostino–Pearson test and by visual inspection of log-transformed quantile–quantile plots. Correlation analyses of functional parameters were performed using Pearson’s correlation test since a normal distribution could be assumed. Functional RV parameters, scan time, and image quality were compared by Wilcoxon’s non-parametric rank-sum test. Bland–Altman plots and linear regression analyses were applied for the comparison of functional values during RMB, SB, and FB by both readers. Inter- and intra-reader agreement was determined by calculating intraclass correlation coefficients (ICCs) for functional results and weighted kappa statistics for subjective quality assessment. A value of 0.01–0.2 indicated slight agreement, 0.21–0.40 indicated fair agreement, 0.41–0.60 indicated moderate agreement, 0.61–0.80 indicated good agreement, and >0.8 indicated excellent agreement [21]. Significance was accepted for *p*-values < 0.05. Statistical analyses were performed using SPSS Statistics software (version 21, SPSS Inc./IBM, Chicago, IL, USA), and GraphPad Prism software (version 6, GraphPad Software, La Jolla, CA, USA).

## 3. Results

Twenty children (50% female, 13.6 ± 3.6 years old, body mass index 20.1 ± 7.2 kg/m^2^, body surface area 1.4 ± 0.4 m^2^) with CHD were analyzed. 

**Figure 1:**
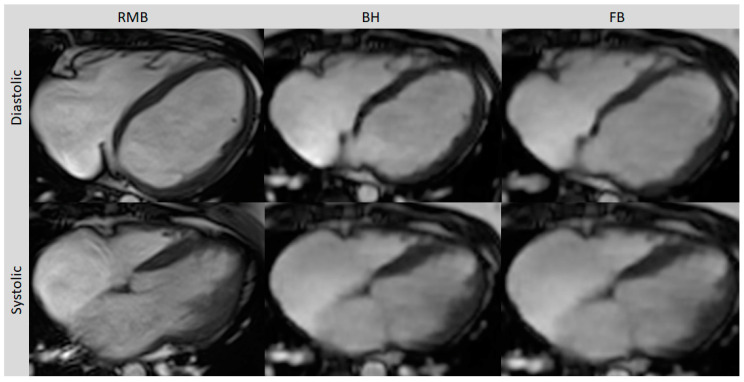
Axial slices of all three cine sequences in systolic and diastolic phases. FB and BH images are almost equivalent to RMB in the delineating blood–myocardium boundary but with typically a slightly blurrier aspect. Retrospective segmented multi-breath hold (RMB), real-time single/double breath hold (BH), real-time free breathing (FB).

**Figure 2:**
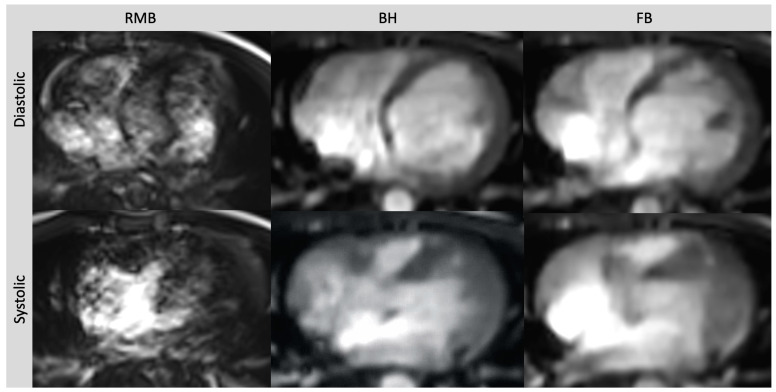
Axial slices of all three cine sequences in systolic and diastolic phases in a patient with severe arrhythmia. Severe artifacts in RMB rendering volumetric evaluation insufficient. Image quality in FB and BH images is reduced but still diagnostic. Retrospective segmented multi-breath hold (RMB), real-time single/double breath hold (BH), real-time free breathing (FB).

Comparing all three methods, the best image quality was provided by RMB (4.5; range 2 to 5) compared to BH (3.9; range 3 to 5; *p* = 0.04) and FB (3.6; range 3 to 5; *p* < 0.01). The image quality with BH was slightly better than with FB but the difference did not reach statistical significance (*p* = 0.07). The diagnostic confidence was comparable between RMB and BH (*p* = 0.65) and between RMB and FB (*p* = 0.84). With RMB, 90% of the cases were rated with high, 5% with intermediate, and 5% with low confidence, whereas with CS-BH and CS-FB, 80% and 75% were rated with high confidence, respectively, and 20% and 25% with intermediate confidence, respectively. Poor image quality with RMB was due to artifacts caused by arrhythmia, whereas BH and FB delivered diagnostic-level image quality in this case (Table 2). Representative images from RMB, BH, and FB are shown In Figure 1 and Figure 2.

**Figure 3:**
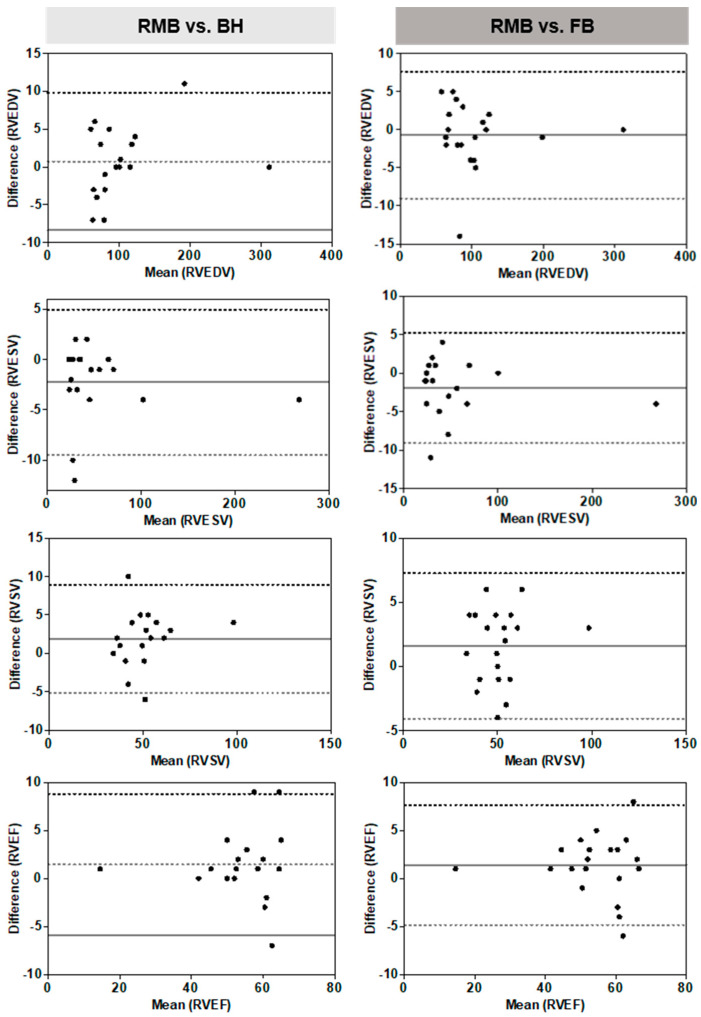
Bland–Altman plots for right ventricle (RV) functional parameters illustrating the differences between RMB to BH and FB (Reader 1). Retrospective segmented multi-breath hold (RMB), real-time single/double breath hold (BH), real-time free breathing (FB), end diastolic volume (RVEDV), end systolic volume (RVESV), stroke volume (RVSV), ejection fraction (RVEF). Stroke volumina are given in mL/m^2^, EF in %. (––) Mean difference; (- - -) 95% limits of agreement (i.e., mean ± 1.96 standard deviation (SD)).

The Bland–Altman plots and linear regression analyses showed good agreement of RV functional assessments between RMB and BH, as well as between RMB and FB (Figure 3).

The RVESV and RVSV with BH and FB showed significant alterations for mean differences compared to RMB. No differences were present in RVEDV or RVEF (Table 3). 

The percentage differences among the three acquisition techniques for all functional parameters were less than 5%. Table 4 shows the mean RV functional parameters for both readers.

**Figure 4:**
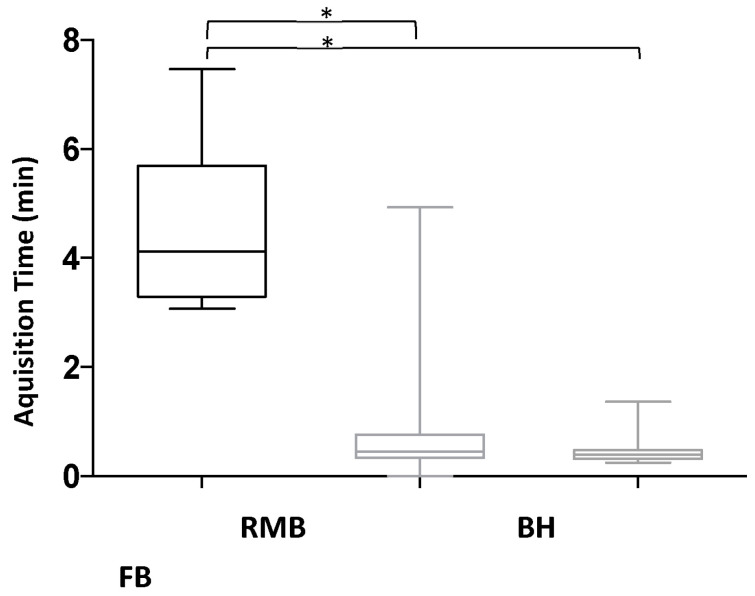
Acquisition time (minutes). Retrospective segmented multi-breath hold (RMB), real-time single/double breath hold (BH), real-time free breathing (FB); * statistically significant (*p* < 0.01).

The scan time was significantly shortened using BH (44 ± 38 s, *p* < 0.01) and FB (24 ± 7 s, *p* < 0.01) compared to RMB (261 ± 68 s). FB acquisition was significantly faster than BH acquisition (*p* < 0.01) (Figure 4).

The inter- and intra-reader variability in RV assessment was low for all three techniques, with excellent intraclass correlations (ICC ≥ 0.93 [range 0.88–0.99]). 

## 4. Discussion

cMRI in patients with congenital heart disease (CHD) remains challenging in routine clinical practice. However, accelerated techniques such as compressed sensing (CS) offer the potential for high-quality imaging with reduced acquisition times, thereby alleviating the burden of cMRI for chronically ill children in the future. 

In our study, we employed real-time CS with breath hold (BH) and free-breathing (FB) protocols to perform cMRI in children with surgically repaired CHD. The results revealed diagnostic image quality that was comparable to that achieved with conventional right ventricular retrospective segmented multi-breath hold balanced steady-state free precession (RMB) acquisition techniques. Although the image quality of the CS techniques was slightly inferior to that of standard RMB acquisition, the BH and FB protocols yielded image quality that was nearly equivalent. Notably, BH and FB consistently produced diagnostic images, whereas RMB failed in a case with arrhythmia. Furthermore, the use of BH or FB significantly reduced the scan time. When assessing right ventricular (RV) parameters, both inter-reader and intra-reader variability were low for BH, CS, and RMB.

In patients without congenital anomalies of the cardiac anatomy, the CS imaging technique has been shown to be accurate and sufficiently reproducible in the evaluation of both LV and RV functional parameters, whereas the subjective image quality has been rated lower compared to RMB [15,16,22,23]. In our study, only small but statistically significant mean differences in RVESV and RVSV values for BH and FB were found compared to RMB. Nevertheless, the differences in all functional parameters were less than 5%, with narrow limits of agreement, and they would be expected to have only minimal effects on clinical decision-making. Importantly, the intra-reader and inter-reader variability of the CS imaging was similar to that of the RMB sequences, indicating reliability for clinical use.

Steeden et al. reported significant differences in RVSV and RVEF parameters when comparing RMB and BH imaging protocols. In their study, the RV functional parameters of pediatric patients were obtained with a short-axis ventricular view [24]. The best orientation (axial or short-axis plane) for functional assessment of the RV is still being debated. Since volumes of the RV seem to conform more closely to the flow parameters of the pulmonary valve in a dedicated axial orientation compared to a short-axis plane [3,16,17], we evaluated the RV functional parameters in an axial orientation. 

The spatial resolution is slightly lower with CS protocols compared to RMB since, to prevent artifacts, the field of view must cover the entire anatomy when using CS [22]. Moreover, the blurrier aspect of CS images could have the potential to impair the unambiguous detectability of epi- and endocardial contours [15,25]. From a clinical point of view, a certain reduction in image quality can be accepted as long as the overall diagnostic value is not limited. No such loss of value was demonstrated in our study. 

In our examination protocols, the RMB images were always acquired at the end of expiration, whereas with FB protocols, the data acquisition was conducted in multiple breathing phases. Since inspiration causes negative intrathoracic pressure, the ventricular functional parameters might be affected as well [26]. However, in our study, this methodical particularity of the CS imaging technique did not impair the reliability of the measurement results.

RMB relies on the regular periodicity of the heart rate to acquire data across multiple cardiac cycles, which are then merged to reconstruct a complete cine slice representing successive heartbeats. However, in the presence of arrhythmia, artifacts can occur due to the reconstruction using data from different phases of the cardiac cycle [27,28]. To mitigate this, arrhythmia rejection algorithms can be applied but may result in exceedingly long breath holds [29]. By incorporating parallel imaging, CS can achieve acceleration rates that enable real-time cardiac cine imaging. This helps to avoid misreferencing of the image data throughout the cardiac cycle [30]. Within our patient collective, we encountered a single patient who experienced non-diagnostic image quality in RMB due to artifacts related to arrhythmia. In this particular patient, ventricular volumetry was not feasible in RMB, whereas both CS sequences consistently provided diagnostic image quality, overcoming the challenges posed by arrhythmia-related artifacts (Figure 2). This is in line with several other studies that have demonstrated the superiority of real-time cine CS MRI images over retrospective gated acquisitions in patients with arrhythmia. In a study conducted by Longère et al. involving a cohort of 71 patients with arrhythmia, the implementation of compressed sensing real-time cine drastically reduced artifacts associated with arrhythmia. As a result, there was a marked improvement in the quality of cine images [29]. In a different study, carried out by Laubrock et al., real-time cine CS imaging was found to enhance the quality of images in 29 patients with atrial fibrillation. Volumetric analysis was feasible, albeit with slightly lower values compared to RMB, and ejection fractions remained comparable [31]. 

Especially in the context of pediatric patients, time is a crucial factor in determining the success of magnetic resonance imaging (cMRI) due to their limited compliance and the frequent need for sedation or general anesthesia during examinations. The ability to obtain high-quality images in a shorter period is highly desirable, as it reduces the potential physical side effects associated with prolonged anesthesia exposure. Moreover, younger patients with CHD often present with unstable clinical conditions, further emphasizing the importance of minimizing scan time to reduce potential risks and complications. A study conducted by Jaimes et al. demonstrated a direct correlation between the duration of the magnetic resonance imaging (MRI) protocol and the success of non-sedated MRI examinations in children aged 1 to 7 years. Specifically, the success rate of studies utilizing protocols with an acquisition time of less than 20 min exceeded 80% [32].

CS examination protocols offer significant advantages in terms of time efficiency compared to conventional methods. In our study, we found that CS sequences utilizing the FB technique allowed for the acquisition of a complete ventricular stack in just 14 s, without any significant loss of image quality. On the other hand, the standard RMB imaging using traditional techniques required a minimum of 3.5 min. This substantial reduction in scan time with CS protocols has the potential to revolutionize the imaging process for children with CHD.

Another noteworthy advantage of CS imaging, specifically with FB protocols, is the ability to overcome the need for artificial ventilation during breath hold examinations. By eliminating these requirements, CS imaging offers improved patient safety and reduces the complexity of the clinical workflow.

Overall, the utilization of CS real-time imaging in children with CHD holds great promise for functional cardiac analysis. The significant time savings, reduced need for sedation or anesthesia, avoidance of artificial ventilation/intubation, and relative insensitivity towards arrhythmia-induced artifacts make CS an attractive option in this patient population. 

### Limitations

Some limitations of the study must be stated. First, the study population was rather small. Second, our cohort did not include many patients with arrhythmia.

## 5. Conclusions

Real-time BH and FB reduce the time to obtain cine cMRI to evaluate RV parameters with sufficient accuracy. CS delivered diagnostic image quality in a significantly shorter examination time compared to RMB imaging protocols. Thus, CS has the potential to replace standard RMB examinations in children with CHD.

## Figures and Tables

**Table 1:** Imaging parameters for cine cardiac MRI. Retrospective segmented multi-breath hold (RMB), real-time single/double breath hold (BH), real-time free breathing (FB), electrocardiogram (ECG), balanced steady-state free precession (bSSFP).

Parameter	RMB	FB	BH
**Orientation**	Transversal	Transversal	Transversal
**Sequence type**	bSSFP	bSSFP	bSSFP
**ECG mode**	Retrospective	Prospective	Prospective
**In plane resolution** **reconstructed (mm)**	1.3 × 1.3	1.6 × 1.6	1.6 × 1.6
**In plane resolution** **acquired (mm)**	1.5 × 1.3	2.1 × 1.6	2.1 × 1.6
**Slice thickness (mm)**	8	8	8
**Section gap (mm)**	2	2	2
**Repetition time (ms)**	45 (interpolated to 25 cardiac phases)	41 (interpolated to 25 cardiac phases)	41 (interpolated to 25 cardiac phases)
**Echo time (ms)**	1.52	1.25	1.25
**Flip angle (degree)**	53	80	80
**Field of view (mm)**			
**Read** **Phase**	210 130%	360 82.10%	360 82.10%
**Image matrix**	177 × 160	143 × 224	143 × 224
**Number of BHs**	10–13 (one slice per breath hold)	0	1–2
**Number of slices**	10–13	10–13	10–13
**Acceleration factor**	GRAPPA factor of 3	Net acceleration factor of 10.2	Net acceleration factor of 10.2
**Bandwidth (Hz/pixel)**	977	859	859
**Number of iterations**	0	50	50
**Total scan time**	261 s Range 130 s–385 s	24 s Range 11 s–27 s	44 s Range 15 s–135 s

**Table 2:** Subjective image quality for right functional analysis in RMB, BH, and FB. Data are presented as number of patients and percentage (n = 20 patients). Retrospective segmented multi-breath hold (RMB), real-time single/double breath hold (BH), real-time free breathing (FB).

	Nondiagnostic	Poor	Adequate	Good	Very Good
RMB	0 (0%)	1 (5%)	1 (5%)	5 (25%)	13 (65%)
BH	0 (0%)	0 (0%)	4 (20%)	13 (65%)	3 (15%)
FB	0 (0%)	0 (0%)	5 (25%)	15 (75%)	0 (0%)

**Table 3:** Difference of absolute right ventricular function parameters between retrospective segmented multi-breath hold (RMB) vs. real-time single/double breath hold (BH) and real-time free breathing (FB) (Reader 1). End diastolic volume (EDV), end systolic volume (ESV), stroke volume (SV), ejection fraction (EF).

Right Ventricle		Difference	
EDV [mL/m^2^]	RMB vs. BH	0.7 ± 5.5	*p* < 0.52
	RMB vs. FB	0.1 ± 5.8	*p* = 0.47
ESV [mL/m^2^]	RMB vs. BH	1.8 ± 5.7	*p* < 0.01
	RMB vs. FB	5.8 ± 12.9	*p* = 0.04
SV [mL/m^2^]	RMB vs. BH	−3.3 ± 7.5	*p* = 0.03
	RMB vs. FB	−3.0 ± 5.9	*p* = 0.03
EF [%]	RMB vs. BH	1.4 ± 3.8	*p* = 0.12
	RMB vs. FB	1.4 ± 3.2	*p* = 0.64

**Table 4:** Right ventricular functional parameters (mean ± SD). Retrospective segmented multi-breath hold (RMB), real-time single/double breath hold (BH), real-time free breathing (FB), end diastolic volume (EDV), end systolic volume (ESV), stroke volume (SV), ejection fraction (EF).

	Reader	RVEDV [mL/m^2^]	RVESV [mL/m^2^]	RVSV [mL/m^2^]	EF [%]
RMB	Reader 1	104.1 ± 56.7	52.1 ± 52.8	51.8 ± 13.8	54.8 ±11.6
	Reader 2	103.9 ± 54.5	52.9 ± 52.4	51.4 ± 13.7	54.3 ± 12.2
BH	Reader 1	104.4 ± 58.4	55.6 ± 55.7	49.8 ± 13.8	53.1 ± 11.9
	Reader 2	105.6 ± 60.0	55.3 ± 55.3	50.6 ± 13.1	53.3 ± 11.7
FB	Reader 1	104.8 ± 56.8	54.0 ± 54.6	50.3 ± 13.4	53.4 ±11.7
	Reader 2	104.5 ± 57.3	53.9 ± 52.5	50.6 ± 13.8	53.8 ± 11.5

## Data Availability

The data supporting the findings of this study are available at reasonable request from the corresponding author. Please note, however, that we are not allowed to make the complete image datasets publicly available, as they contain personal information that could compromise the study participants’ privacy and consent.

## References

[1] Warnes C.A. (2009). Adult Congenital Heart Disease: Importance of the Right Ventricle. J. Am. Coll. Cardiol..

[2] A Davlouros P., Niwa K., Webb G., A Gatzoulis M. (2006). The right ventricle in congenital heart disease. Heart.

[3] Clarke C.J., Gurka M.J., Norton P.T., Kramer C.M., Hoyer A.W. (2012). Assessment of the Accuracy and Reproducibility of RV Volume Measurements by CMR in Congenital Heart Disease. JACC Cardiovasc. Imaging.

[4] Guihaire J., Haddad F., Mercier O., Murphy D.J., Wu J.C., Fadel E. (2012). The Right Heart in Congenital Heart Disease, Mechanisms and Recent Advances. J. Clin. Exp. Cardiol..

[5] Gallo-Bernal S., Bedoya M.A., Gee M.S., Jaimes C. (2022). Pediatric magnetic resonance imaging: Faster is better. Pediatr. Radiol..

[6] Davidson A.J., Morton N.S., Arnup S.J., de Graaff J.C., Disma N., Withington D.E., Frawley G., Hunt R.W., Hardy P., Khotcholava M. (2015). Apnea after Awake Regional and General Anesthesia in Infants: The General Anesthesia Compared to Spinal Anesthesia Study—Comparing Apnea and Neurodevelopmental Outcomes, a Randomized Controlled Trial. Anesthesiology.

[7] Davidson A.J., Disma N., de Graaff J.C., E Withington D., Dorris L., Bell G., Stargatt R., Bellinger D.C., Schuster T., Arnup S.J. (2015). Neurodevelopmental outcome at 2 years of age after general anaesthesia and awake-regional anaesthesia in infancy (GAS): An international multicentre, randomised controlled trial. Lancet.

[8] McCann M.E., de Graaff J.C., Dorris L., Disma N., Withington D., Bell G., Grobler A., Stargatt R., Hunt R.W., Sheppard S.J. (2019). Neurodevelopmental outcome at 5 years of age after general anaesthesia or awake-regional anaesthesia in infancy (GAS): An international, multicentre, randomised, controlled equivalence trial. Lancet.

[9] Flick R.P., Katusic S.K., Colligan R.C., Wilder R.T., Voigt R.G., Olson M.D., Sprung J., Weaver A.L., Schroeder D.R., Warner D.O. (2011). Cognitive and Behavioral Outcomes After Early Exposure to Anesthesia and Surgery. Pediatrics.

[10] Christopher A.B., Quinn R.E., Zoulfagharian S., Matisoff A.J., Cross R.R., Xue H., Campbell-Washburn A., Olivieri L.J. (2020). Motion-corrected cardiac MRI is associated with decreased anesthesia exposure in children. Pediatr. Radiol..

[11] Artunduaga M., Liu C.A., Morin C.E., Serai S.D., Udayasankar U., Greer M.-L.C., Gee M.S. (2021). Safety challenges related to the use of sedation and general anesthesia in pediatric patients undergoing magnetic resonance imaging examinations. Pediatr. Radiol..

[12] Axel L., Otazo R. (2016). Accelerated MRI for the assessment of cardiac function. Br. J. Radiol..

[13] Maceira A.M., Prasad S.K., Khan M., Pennell D.J. (2006). Reference right ventricular systolic and diastolic function normalized to age, gender and body surface area from steady-state free precession cardiovascular magnetic resonance. Eur. Heart J..

[14] Keenan N.G., Pennell D.J. (2007). CMR of Ventricular Function. Echocardiography.

[15] Treutlein C., Wiesmüller M., May M.S., Heiss R., Hepp T., Uder M., Wuest W. (2019). Complete Free-breathing Adenosine Stress Cardiac MRI Using Compressed Sensing and Motion Correction: Comparison of Functional Parameters, Perfusion, and Late Enhancement with the Standard Breath-holding Examination. Radiol. Cardiothorac. Imaging.

[16] James S.H., Wald R., Wintersperger B.J., Jimenez-Juan L., Deva D., Crean A.M., Nguyen E., Paul N.S., Ley S. (2013). Accuracy of Right and Left Ventricular Functional Assessment by Short-Axis vs. Axial Cine Steady-State Free-Precession Magnetic Resonance Imaging: Intrapatient Correlation with Main Pulmonary Artery and Ascending Aorta Phase-Contrast Flow Measurements. Can. Assoc. Radiol. J..

[17] Van Der Bom T., Romeih S., Groenink M., Pieper P.G., Van Dijk A.P., Helbing W.A., Zwinderman A.H., Mulder B.J., Bouma B.J. (2014). Evaluating the Systemic Right Ventricle by Cardiovascular Magnetic Resonance: Short Axis or Axial Slices?. Congenit. Heart Dis..

[18] Sudarski S., Henzler T., Haubenreisser H., Dösch C., Zenge M.O., Nadar M.S., Borggrefe M., Papavassiliu T., Schmidt M., Schoenberg S.O. (2017). Free-breathing Sparse Sampling Cine MR Imaging with Iterative Reconstruction for the Assessment of Left Ventricular Function and Mass at 3.0 T. Radiology.

[19] Alfakih K., Plein S., Bloomer T., Jones T., Ridgway J., Sivananthan M. (2003). Comparison of right ventricular volume measurements between axial and short axis orientation using steady-state free precession magnetic resonance imaging. J. Magn. Reson. Imaging.

[20] El Edelbi R., Lindemalm S., Eksborg S. (2012). Estimation of body surface area in various childhood ages—Validation of the Mosteller formula. Acta Paediatr..

[21] Klinke V., Muzzarelli S., Lauriers N., Locca D., Vincenti G., Monney P., Lu C., Nothnagel D., Pilz G., Lombardi M. (2013). Quality assessment of cardiovascular magnetic resonance in the setting of the European CMR registry: Description and validation of standardized criteria. J. Cardiovasc. Magn. Reson..

[22] Vincenti G., Monney P., Chaptinel J., Rutz T., Coppo S., Zenge M.O., Schmidt M., Nadar M.S., Piccini D., Chèvre P. (2014). Compressed Sensing Single–Breath-Hold CMR for Fast Quantification of LV Function, Volumes, and Mass. JACC Cardiovasc. Imaging.

[23] Haubenreisser H., Henzler T., Budjan J., Sudarski S., Zenge M.O., Schmidt M.R., Nadar M.S., Borggrefe M., Schoenberg S.O., Papavassiliu T. (2016). Right Ventricular Imaging in 25 Seconds: Evaluating the Use of Sparse Sampling CINE With Iterative Reconstruction for Volumetric Analysis of the Right Ventricle. Investig. Radiol..

[24] Steeden J.A., Kowalik G.T., Tann O., Hughes M., Mortensen K.H., Muthurangu V. (2018). Real-time assessment of right and left ventricular volumes and function in children using high spatiotemporal resolution spiral bSSFP with compressed sensing. J. Cardiovasc. Magn. Reson..

[25] Feng C., Zhang S., Zhao D., Li C. (2016). Simultaneous extraction of endocardial and epicardial contours of the left ventricle by distance regularized level sets. Med. Phys..

[26] Ogilvie L.M., Edgett B.A., Gray S., Al-Mufty S., Huber J.S., Brunt K.R., Simpson J.A. (2021). A new approach to improve the hemodynamic assessment of cardiac function independent of respiratory influence. Sci. Rep..

[27] Lenz G.W., Haacke E.M., White R.D. (1989). Retrospective cardiac gating: A review of technical aspects and future directions. Magn. Reson. Imaging.

[28] Madore B., Hoge W.S., Chao T.-C., Zientara G.P., Chu R. (2011). Retrospectively gated cardiac cine imaging with temporal and spatial acceleration. Magn. Reson. Imaging.

[29] Longère B., Allard P.-E., Gkizas C.V., Coisne A., Hennicaux J., Simeone A., Schmidt M., Forman C., Toupin S., Montaigne D. (2021). Compressed Sensing Real-Time Cine Reduces CMR Arrhythmia-Related Artifacts. J. Clin. Med..

[30] Feng L., Srichai M.B., Lim R.P., Harrison A., King W., Adluru G., Dibella E.V.R., Sodickson D.K., Otazo R., Kim D. (2012). Highly accelerated real-time cardiac cine MRI using *k*-*t* SPARSE-SENSE. Magn. Reson. Med..

[31] Laubrock K., von Loesch T., Steinmetz M., Lotz J., Frahm J., Uecker M., Unterberg-Buchwald C. (2022). Imaging of arrhythmia: Real-time cardiac magnetic resonance imaging in atrial fibrillation. Eur. J. Radiol. Open.

[32] Jaimes C., Robson C.D., Machado-Rivas F., Yang E., Mahan K., Bixby S.D., Robertson R.L. (2021). Success of Nonsedated Neuroradiologic MRI in Children 1–7 Years Old. Am. J. Roentgenol..

